# Supplementary material for: Are carbon emissions trading and green financial instruments synergistic? -Comprehensive quantitative research based on content analysis
Source: PLoS One. 2024 Mar 7;19(3):e0298601. doi: 10.1371/journal.pone.0298601 (PMC10919601; doi:10.1371/journal.pone.0298601)
Supplement: S1 Data — (ZIP) [file pone.0298601.s001.zip › Data/地级市Z_F工作报告.zip!/░▓╗╒/╦▐╓▌╩╨/└┤╫╘╣1⁄2╓┌║┼í╛┬φ┐╦╩2╛▌═°í┐_2017.docx]

2017年1月11日在宿州市第四届人民代表大会第六次会议上

市委副书记、代市长 杨军

各位代表：

现在，我代表市人民政府，向大会报告政府工作，请予审议，并请市政协委员和其他列席人员提出意见。

一、“十三五”实现良好开局

过去一年，在省委、省政府和市委的坚强领导下，全市上下全面贯彻党的十八大和十八届三中、四中、五中、六中全会精神，深入贯彻习近平总书记系列重要讲话特别是视察安徽重要讲话精神，攻坚克难，开拓进取，较好完成了市四届人大五次会议确定的目标任务。

初步核算，全市生产总值1351.8亿元，增长9.1%；财政收入139.8亿元，增长11.3%；固定资产投资1270亿元，增长12.1%；社会消费品零售总额476.9亿元，增长12.3%；城镇、农村常住居民人均可支配收入25533元和9917元，分别增长8.1%、8.5%；三次产业结构持续优化，一产比重首次降至20%以下；节能减排达到省控目标；主要动态指标继续保持全省前列。特别是县域经济发展加快，主要经济指标增速高于全市平均水平，经济总量占全市比重达到85%，整体实力显著提升。

一是产业转型步伐加快。工业主导地位突出，规模以上工业增加值405.6亿元，增长9.3%；规模以上工业企业1281户，产值超亿元企业530户。持续实施“3111”工程，“三个10”项目顺利推进，实现首位产业产值720亿元。“调转促”步伐加快，实施传统产业改造升级，完成技术改造投资430亿元，增长15%；依托“全创改”，统筹推进“三重一创”，云计算产业基地入选省级战略性新兴产业集聚发展基地，市经开区生物医药、砀山果蔬食品生物科技、萧县功能性新材料3个市级战新基地启动建设，全市战略性新兴产业产值、高新技术产业工业增加值分别增长29.6%、25.9%。3D打印、石墨烯研究院等创新平台相继建立，国盾量子通信落户宿州，新增省级以上研发机构2家。产业发展后劲增强，钱营孜低热值煤发电、和泰陶瓷等190个项目开工建设，新亚电子等192个项目进度加快，立诚包装等85个项目建成投产，“大新专”项目投资640亿元，完成年度计划的146%，获批专项建设基金29.8亿元。积极对接徽商大会等招商活动，实际到位内资1040亿元，增长15.6%；利用外资7.3亿美元，增长8%。新型业态加速成长，电子商务异军突起，全市网上农产品销售55亿元、增长20%，泗县获评国家级电子商务进农村综合示范县。砀山、灵璧分别入选国家全域旅游示范区、全国休闲农业与乡村旅游示范县，萧县获评中国最佳乡村旅游目的地，全市旅游总收入120亿元，增长20.7%。

二是城乡建设面貌一新。城市总体规划修编顺利启动，《大宿城空间战略发展规划》编制积极推进。全市实施重点工程205项，完成投资231.9亿元。郑徐高铁宿州段、宿灵泗快速通道正式通车，宿萧砀快速通道基本建成。宿州东站扩建、市政务服务中心加快建设，拂晓大道综合改造顺利完成，万达广场、南翔云集开业运营，植物园建成开放，主城区实现双“80”目标。“555”工程扎实推进，县区开工建设重点项目99个，渔沟镇入选首批省级特色小镇。棚户区改造加快推进，建设房产超市，充实房源项目库，货币化安置比例达70%；完成征迁拆违733万平方米，新开工各类保障房2.2万套，开工率、基本建成率均居全省前列。城市管理文明有序，争创全国文明城市活动扎实开展，荣获国家园林城市称号，“城管服务超市”入选全国创新社会治理典型案例。城市功能持续完善，“智慧城市”13个平台类、民生类项目一体推进，成功获批国家首批电信普遍服务试点城市、“宽带中国”示范城市、省首批优先发展公交示范城市。城乡生态不断改善，全市新增绿地面积487万平方米，建成绿道70公里，新造林14.7万亩，森林增长工程通过验收，新汴河景观带获批国家级水利风景区，石龙湖国家湿地公园正式命名，埇桥区获评全国绿化模范县区；美丽乡村建设加快，完成41个乡镇政府驻地建成区整治、43个中心村建设任务，“三线三边”环境治理成效显著；环境执法检查力度加大，非煤矿山整治有力推进，秸秆综禁成效明显，大气和水污染防治取得阶段性成果。宿州变得更美了，为宿州点赞的更多了。

三是现代农业全面提升。粮食总产403.2万吨，实现“十三连丰”。建设创新型农业现代化先行区，着力打造现代农业“两区”升级版。农业经营主体向多元转变，现代农业产业化联合体、家庭农场、农民专业合作社分别达到229个、3970个、9893个，分别增长30.9%、9%、11.5%。农业产业化综合水平全省领先，规模以上农产品加工企业完成加工产值1027.8亿元、增长9.2%，市级以上龙头企业516家。砀山酥梨进入“全国果品业最具投资价值品牌30强”。农村重点改革扎实推进，土地承包经营权确权登记颁证顺利实施，农业规模经营面积占耕地比重达25%，“劝耕贷”试点在全国推广。病险水库水闸除险加固、中小河流治理等重点水利工程加快推进，新增高效节水灌溉面积1万亩，埇桥区荣获省“江淮杯”四连冠。解决20万人饮水安全问题。主要农作物耕种收综合机械化水平达87%以上。

四是脱贫攻坚稳步推进。11.91万贫困人口、140个贫困村脱贫出列。建立健全“1+28”政策体系，形成脱贫攻坚政策“组合拳”。强化定点包保责任，359个贫困村实现“脱贫攻坚第一书记”全覆盖。落实精准识别和动态管理机制，扶贫对象基础信息进一步完善。启动脱贫攻坚“十大工程”，5000个产业扶贫项目精准到户，建成12100个户用、189个村级光伏电站。在全省率先开展贫困人口综合医保“一站式”即时结算，市内医疗机构就诊费用通过综合补偿后，其余合规费用再补助90%，在全国健康脱贫工作会上作经验介绍。实施易地扶贫搬迁3700人，完成贫困户危房改造8150户，建档立卡贫困家庭高中生学杂费全部免除。

五是改革开放持续深入。供给侧结构性改革扎实推进，“去降补”任务落地见效，商品房去化周期由16.5个月降至12个月，政府平台融资成本不超过银行贷款基准利率的1.3倍，“营改增”降低企业税负3亿元，减负率25%。“放管服”改革深入实施，行政审批“三集中、三到位”全面落实，行政审批类、公共服务类项目“网上可办”；大众创业、万众创新深入推进，“五证合一、一照一码”全面实施，新登记注册企业10870户，增长29%。金融服务能力不断增强，全年新增贷款166亿元、增长38%，直接融资102亿元、增长308%，均创历史新高。主板辅导备案企业2家，新三板挂牌企业4家。大力发展股权债权投资基金，各类基金总规模93亿元。萧县民间投资工作在国务院大督查中获得肯定。城市智能管网、206国道北段改建工程等6个PPP项目有序推进，吸引社会资本44亿元。户籍制度改革稳步推进，新增城镇落户8667人，农村转移人口市民化步伐加快。基本实现不动产统一登记全域发证。文化体制、医药卫生、国资国企、统计调查等重点领域改革有序推进。宿州海关正式开关，全市进出口总额4.7亿美元。园区发展活力增强，市经开区成功扩区，宿马园区荣膺“中国产业园区营商环境百强”，市高新区蝉联“中国产业园区创新力百佳”，鞋城成功举办第五届中国制鞋产业发展高层论坛。

六是民生福祉不断改善。全市民生支出265亿元，占财政总支出的85.1%。33项民生工程全面完成，再获全省民生工程绩效奖补先进市。10件惠民实事顺利实施，新增电动公交车60辆，新建改建乡镇公厕100座，市、县城区老旧电线改造稳步推进。新增城镇就业5.4万人，城镇登记失业率控制在4.5%以内。“五险”参保180万人次，建立机关事业单位工作人员养老保险制度，企业退休人员基本养老金人均月增150.2元，城乡低保人均月保障标准分别增长11.6%、36.4%，农村五保供养标准增长33%。新建改扩建公办幼儿园55所，完成义务教育学校标准化建设155所，4所职教中心转为普通中专。埇桥区获评全国社区教育示范区。二级以上医疗机构增加到24所，创建国家级群众满意乡镇卫生院16个、省级群众满意村卫生室11个。实施全面两孩政策，人口计生工作保持全省前列，泗县获评全国计划生育优质服务县。入选“中国好人”5名、“安徽好人”25名。《平安是福》晋京赴省演出圆满成功。成功举办2016宿州国际网球公开赛和第二届宿州国际半程马拉松赛。食品药品监管规范有序，基层监管所标准化建设走在全省前列。安全生产监管执法推进年活动深入开展，依法关闭非法企业418家，取缔非法加油站614个，安全生产形势总体稳定。“平安宿州”建设扎实推进，应急管理体系不断完善。“七五”普法和第三次全国农业普查顺利启动。全民国防教育、国防动员、人民防空工作进一步加强，全国双拥模范城实现“七连冠”。全国无偿献血先进城市实现“八连冠”，侨务、科普、防震减灾等工作荣获全国先进荣誉称号，物价、外事、对台、档案、气象、民族宗教、妇女儿童、地方志、残疾人等事业得到进一步加强。

在统筹做好各项工作的同时，我们始终高度重视政府自身建设。扎实开展“两学一做”学习教育，积极开展“讲看齐、见行动”学习讨论，严格落实中央八项规定精神和省市“30条”，持续深化效能建设，有效整治“中梗阻”和“基层板结”。深入推进依法行政，落实重大决策法定程序，推进合法性审查机制全覆盖，加强和改进政府立法，提请市人大常委会审议地方性法规2件。自觉接受人大法律监督、政协民主监督和社会舆论监督，办理人大代表议案和建议121件，政协建议案和委员提案252件。深化政务公开，切实做好市长热线和网络问政工作，一批群众关注的热点问题得到有效解决。认真履行政府系统全面从严治党责任，落实中央巡视组“回头看”反馈问题整改，加强行政监察和审计监督，严肃查处违纪违法案件，党风廉政建设和反腐败斗争取得新成效。

各位代表！一年来的实践探索，我们对推动宿州发展有了更加深刻的体会：

必须坚持看齐在前，以习近平总书记系列重要讲话精神为指引，做好结合转化文章，着力释放自身发展潜力；

必须坚持发展至上，把工业化、城镇化“双轮驱动”作为强市基本方略，持续推进工业“3111”和城乡建设“555”工程，着力夯实跨越赶超基础；

必须坚持民生为本，把增进人民福祉作为一切工作的出发点和落脚点，扎实推进脱贫攻坚，着力保障改善民生；

必须坚持担当有为，持之以恒抓主抓重、苦干实干、提速提效、争先争优，着力营造风清气正、干事创业的优良环境。

奋斗历尽艰辛，成绩来之不易。这是市委总揽全局、坚强领导的结果，是市人大、市政协和社会各界监督支持的结果，是全市上下同心协力、顽强拼搏的结果。在此，我代表市人民政府，向奋战在各行各业的全体劳动者，向离退休老同志，向各民主党派、工商联和人民团体，向中央和省驻宿单位、人民解放军驻宿部队、武警官兵、政法公安干警，向参与宿州建设的海内外客商、广大援宿干部，以及所有支持宿州发展的各界人士，表示衷心的感谢，并致以崇高的敬意！

在肯定成绩的同时，我们清醒认识到，我市发展中仍存在不少问题和不足：实体经济困难依然较多，企业生产经营成本较高，制造业投资、民间投资有所回落；新兴产业规模较小，现代服务业发展不足，人才相对缺乏，创新驱动能力不强；城镇化水平较低，中心城区能级不强，城乡县区之间发展不均衡；脱贫攻坚任务艰巨，环境保护、社会保障和公共服务均等化水平与群众期盼还有不小差距；政府职能转变尚不到位，少数干部不敢为、不愿为、不善为现象不同程度存在，发展环境仍需进一步优化。对此，我们将高度重视，认真解决，绝不辜负全市人民的期望和重托！

二、以五大发展理念统领发展全局

今年是党的十九大召开之年，是实施“十三五”规划的重要一年，也是推进供给侧结构性改革的深化之年。当前，经济发展新常态的特征更加明显，长期向好基本面没有改变。经过多年努力，我市经济总量、产业素质、综合实力都有了较大提升，新型工业化、城镇化和农业现代化潜力巨大，政策叠加效应逐步显现，改革红利持续释放，奠定了实现新发展的坚实基础。只要我们坚定信心，抢抓机遇，迎难而上，就一定能够实现“中部崛起闯新路、皖北振兴走前列”的奋斗目标。

立足新起点，面对新征程，省第十次党代会、市第五次党代会吹响了全面实施五大发展行动计划、坚定不移闯出新路的战斗号角，接下来必须真抓实干，更加坚定地用新发展理念统领发展全局。突出创新发展，坚定不移下好创新先手棋，以持续创新推动经济发展不断迈上新台阶；突出协调发展，促进城乡发展要素平等转换、合理配置，加快建成城乡区域一体发展新体系；突出绿色发展，坚持绿水青山和金山银山有机统一，把生态文明建设融入经济社会发展全过程；突出开放发展，加快建设省际交汇区域中心城市，着力打造皖北改革开放新高地；突出共享发展，坚持以人民为中心的发展思想，坚决打赢脱贫攻坚战，切实增进人民群众的获得感、幸福感。

今年工作的总体要求是：全面贯彻党的十八大和十八届三中、四中、五中、六中全会精神，深入贯彻习近平总书记系列重要讲话特别是视察安徽重要讲话精神，坚持稳中求进工作总基调，牢固树立和贯彻落实新发展理念，适应把握引领经济发展新常态，以提高发展质量和效益为中心，以推进供给侧结构性改革为主线，以实施五大发展行动计划为总抓手，深入实施新型工业化、城镇化双轮驱动，着力抓好脱贫攻坚和民生保障，加快振兴县域经济，统筹推进各项工作，加快建设五大发展的美好宿州。

经济社会发展主要预期目标是：全市生产总值增长9%左右，财政收入保持同步增长，固定资产投资增长12%左右，规模以上工业增加值增长9.5%左右，社会消费品零售总额增长12%左右，城镇、农村常住居民人均可支配收入分别增长8.5%、9.5%，城镇登记失业率控制在4.5%以内，节能减排完成年度任务，主要动态指标继续位居全省前列。重点做好以下五个方面工作：

（一）突出创新驱动，持续抓好工业经济

加快产业发展。实施第二个“3111”工程，完成“三个10”任务，各县区、园区分别新签约规上工业项目10个、新开工规上工业项目10个、新投产规上工业项目10个，实现规模以上工业增加值440亿元。壮大战略性新兴产业，统筹推进“三重一创”，做大做强做实省、市两级战新产业集聚发展基地，推进市经开区生物医药产业园申报省级战新产业集聚发展基地，择优再启动一批市级基地。着力推进高新技术产业发展，高新技术企业达到100家。力争战略性新兴产业产值、高新技术产业工业增加值分别增长30%、16%。深入实施中国制造2025宿州篇，大力推进“互联网+”行动，全面提升轻纺鞋服、食品加工、化工建材、板材家居等传统产业。实施重点技改“1245”行动计划，推进重点技改项目200个，完成技改投资470亿元。开展质量品牌提升工程，争创一批省名牌产品、国家地理标志产品，创建国家质量强市示范市。

主攻招商引资。坚持“工业项目、招大引强、税源企业和环境保护”原则，推进战略招商、产业招商、全员招商、以商招商，实际到位内资1160亿元、增长12%，实际利用外资增长7%。坚持招商周、领导带头招商制度，调动各类主体招商积极性，真招商、招真商。全面落实“四督四保”要求，力促美为食品等158个项目开工，加快紫金新材料、和佳医疗等246个项目进度，确保海升果酒、吉之宝凉茶等67个项目建成投产，完成固定资产投资1420亿元、工业投资750亿元。新谋划亿元以上项目不少于300个，其中5亿元以上项目150个。不断充实“大新专”项目库，确保重点项目储备规模超5000亿元。加大引智引技力度，引进高层次科技创新团队5个、创新创业人才20名。大力发展民营经济，力争民营经济增加值占GDP比重达到65%。

夯实园区载体。引导各类园区依法合规、节约集约、错位发展，壮大主导产业，力争省级开发区规模以上工业增加值占全市比重超过30%。支持市经开区发展生物医药产业，支持宿马园区引进一批能够迅速集聚人气的工业项目，支持市高新区壮大云计算产业，支持鞋城产业集聚和生产配套功能提升。推动各县区开发区发展，加快砀山通用航空工业园、张江萧县高科技园、埇桥宿州绿色家居产业园、灵璧医药健康产业园、泗县当涂现代产业园建设。推进产城融合发展，加快水、电、气、路等基础设施和生活服务设施建设，完善园区配套功能，提升项目承载能力和吸纳就业能力。

提升创新能力。大力实施创新驱动发展战略，系统推进“全创改”，加快构建科技创新创业孵化体系。认真贯彻省“1+6+2”政策和我市支持科技创新实施办法，研究制定相关配套政策。支持企业申报重点实验室、工程技术研究中心，培育省级以上企业技术中心3家、科技企业孵化器3万平方米。深化产学研协同创新，新增院士工作站2家。各县区、园区结合自身产业特点，与高校、中科院研究所建立合作关系，围绕各自首位产业分别打造一个“双创”发展平台。积极对接国家和省各类创新基金，用好用活科技投入政策，确保全市研发经费投入5亿元，新增发明专利授权量215件。全面落实股权激励和技术入股有关所得税优惠政策，加强知识产权保护，促进更多的科技成果在我市开花结果。

（二）突出城乡统筹，持续推进新型城镇化

掀起棚改热潮。抢抓新型城镇化试点机遇，加快棚户区改造步伐，围绕“五年规划、三年完成”目标，完成棚户区改造1500万平方米以上，累计完成总任务的80%。坚持市区联动，全面拉开道东区域棚改序幕，改善道东区域形象。大力推进棚改货币化安置，货币化安置率60%以上。加大非住宅商品房去库存力度，加快建设房产超市。有序实施老旧小区改造，不断改善群众居住条件，同步推进安置房配套基础设施建设。

加快城乡建设。投资300亿元，实施四大类210项重点工程。主城区力争实现“双85”目标，产城一体推进汴北发展，启动电视发射塔和各类体育场馆建设，加快市立医院新区、动物园建设，全面建成市客运中心站，确保儿童医院、体育馆、城市规划展示馆、“三馆”投入使用。继续实施“555”工程。县城5项工程：规划实施市级及以上特色小镇不少于3个；提升园林绿化工程不少于10个；完善停车场、一类公厕、充电站等公共服务设施不少于10个；建设民生项目周边道路等配套基础设施不少于10个；累计完成棚户区改造总任务的80%。县城5项任务：完成PPP融资5亿元；征迁整理净地1000亩；完成补充耕地指标3200亩；完成高标准基本农田建设省下达任务；完成县级农村垃圾治理和污水治理专项规划。镇村5项工作：创建1家省级优秀旅游乡镇或省级特色景观旅游名镇；贯彻实施市农村垃圾治理条例，实现农村生活垃圾治理全覆盖；创建1个年经营性收益50万元以上的集体经济“示范村”；消除年经营性收益2万元以下的集体经济“空壳村”达上年基数20%以上；完成辖区50%以上县镇政府驻地生活污水管网、处理设施建设和非卫生厕所改造。

完善基础设施。启动宿州火车站综合改造，力争完成符夹线扩能改造、淮北至萧县北客车联络线、宿州东站扩建、宿淮铁路蒿沟站建设，持续推进淮宿蚌城际铁路、合肥-新沂高铁、宿州-蚌埠高速、宿州-阜阳高速宿州段前期工作。支持萧县轨道交通对接徐州地铁一号线。开工建设符离大道、宿淮快速通道等8条国省干线一级公路。推进宿州机场、泗县通用机场前期工作，开工建设砀山通用机场。完善城市路网体系，加快建设西外环线，完成主城区道路交口渠化规划编制。实施银河三路综合改造，加快人民路与拂晓大道地下综合管廊建设。加快城区夜景照明等PPP项目建设。加快浍河航道升级改造和新汴河复航综合整治前期工作。全面完成农村电网改造升级工程。继续实施“宽带宿州”战略，加快“无线城市”建设。

提升城市管理。持续开展争创全国文明城市活动，不断提升城市管理精细化水平。加快“智慧城市”建设，抓好信息资源整合共享，推动互联网、大数据与城市管理深度融合。大力实施海绵城市、城市绿道、花满宿州建设，全市新增提升绿地800万平方米以上，新建绿道70公里，建设街头游园20个。加快推进公交示范城市建设。落实市容治理条例，开展市容市貌综合整治，完善污水、垃圾、供水等配套设施，提升城市服务功能。积极推进城市执法体制改革，改进城市管理工作，促进共建共管、共建共享。

（三）突出三产融合，持续振兴县域经济

加快建设创新型农业现代化先行区。推进农业供给侧结构性改革，全面落实“五个三”要求，推动一二三产融合发展。深入实施“4636”行动计划，加快县区核心区、乡镇示范园建设。推进多种形式适度规模经营，市级现代农业产业化联合体发展到250个，新增示范家庭农场、示范农民合作社各80个。提高农业装备和技术水平，推进农业标准化生产，积极发展品牌农业、特色农业和绿色农业，提升农产品供给质量。积极稳妥推进农村综合改革，抓好农村土地承包经营权确权登记颁证扫尾工作，扩大农村承包土地经营权抵押贷款试点，加快农村产权流转交易市场和仲裁机构建设。推进资源变资产、资金变股金、农民变股东“三变”改革和农村集体资产股份合作制改革，每个乡镇选择一个村开展“三变”改革试点。加强重点水利工程建设，开工怀洪新河水系洼地治理工程，汛前完成2条中小河流治理。加快返乡创业园等载体建设，促进农民工返乡就业创业。完成第三次全国农业普查，摸清“三农”新家底。

全力打好现代农业“三大战役”。推进现代农业产业化，扶持龙头企业做大做强，实现农产品加工产值1100亿元，市级以上龙头企业突破600家，每个县区新增产值超10亿元农业产业化企业不少于1家。推进秸秆综合利用，鼓励社会资本建立秸秆收储和加工处理设施，规划建设秸秆收储点30个。做大做强淮宿建材等企业，加快安徽禾吉等项目建设，确保康桥生物项目建成投产，每个县区各引进亿元以上秸秆综合利用企业不少于1家，全市秸秆产业化利用率每年提高20%。统筹推进美丽乡村、休闲农业和乡村旅游，深入实施“三线三边”环境治理，再建39个美丽乡村示范村，完成48个乡镇政府驻地建成区整改建设，每个县区建成市级休闲农业与乡村旅游示范点2个。

继续主攻县域经济。支持各县区发挥比较优势，培育壮大首位产业，增强县域经济综合实力。继续推进乡镇工业项目向县区开发区集中、农村人口向县城集中、耕地向农业新型经营主体集中，夯实县域发展产业基础。扎实推进镇当县建工作，扶持有条件的乡镇因地制宜发展优势产业。开展“一县一策”专项扶持，强化工作调度，加强督查考核，促进县域经济加快发展，对全市经济的支撑作用更加凸显。

加快提升现代服务业。加快发展科技研发、信息咨询、检验检测等生产性服务业，壮大信息消费、健康养老等新兴服务业，促进生活性服务业向精细化、高品质转变，创建省级服务业集聚区1家。实施“农业+互联网”行动，积极构建农村电子商务体系，努力提高工业品下乡、农产品进城的电商贡献率。加强与国内外知名电商的战略合作，支持建设电商产业园，大力发展跨境电商业务。深入挖掘地方文化资源，促进文化旅游融合发展，抓好皇藏峪5A级景区创建，推进新汴河风景区等4A级景区创建，全力打造符离大道沿线景点，旅游总收入超过130亿元。拓展完善市县乡村四级交通物流网络，新建A级以上物流企业5家。开工建设苏宁广场，加快南翔恒泰商贸物流城、国购广场、宿州百大农产品物流中心三期建设。新增限额以上商贸流通企业70家。

（四）突出问题导向，持续深化改革开放

推进重点改革。围绕基础性、关键性领域，全面落实国家和省级改革试点任务，更好发挥改革牵引作用。加大供给侧结构性改革力度，全面落实“去降补”各项任务。深化财税体制改革，建立全面规范、公开透明的预算制度，加强全口径政府债务管理。持续推进“放管服”和“三集中、三到位”等行政管理体制改革，不断提高行政效能。统筹推进户籍制度改革，全面落实“五权不变、五有并轨”，加快农业转移人口市民化进程。深化商事制度改革，优化营商环境。加快社会信用体系建设，推进信用信息互联共享。积极稳妥推进教育、文化、卫生、司法、国资国企、社会治理等领域改革，使改革更好对接发展所需、民心所向。

强化金融支撑。推进金融和资本创新体系建设，继续实施金融“五个一工程”，全年新增贷款155亿元、增长15%，实现直接融资55亿元。充分利用贫困县上市挂牌优惠政策，力争主板在审2家、辅导备案2家，新增新三板挂牌5家。深化投融资体制改革，培育壮大市级投融资服务平台，继续做好专项建设基金争取工作，积极推广PPP模式，引导社会力量参与重点领域建设。积极引进股份制商业银行来宿设立分支机构，深入推进“4321”新型政银担合作、“税融通”贷款、续贷过桥等金融创新业务。做大各类基金规模，加快发展天使投资基金、风险投资基金、新三板股权投资基金等新兴金融业态。完善金融风险监测预警机制，加强金融风险管控，打击非法金融活动，守住风险底线。

扩大对外开放。积极融入中部崛起、淮河生态经济带、皖北振兴等国家和省重大战略，以大开放推动大发展。加强区域合作交流，推进宿州与淮北一体化发展、与徐州全面融合发展，主动参与淮海经济区核心区一体化建设，提升基础设施和市场体系互通对接能力。加快推进大通道大平台大通关建设，积极申报建设保税物流中心，完善“一站式”通关服务体系，加快建设内陆无水港。大力发展外向型经济，引导有实力的企业开展对外合作，全市进出口增幅高于全省平均水平。

（五）突出民生福祉，持续完善社会建设

扎实推进绿色发展。牢固树立绿水青山就是金山银山的发展理念，坚持生态优先，严格环境执法监管，深入实施大气、水、土壤污染防治行动计划。持续抓好非煤矿山整治，强化燃煤、扬尘等污染控制，加强环境空气质量监测能力建设，确保完成空气质量考核目标。积极建设黄河故道等“两河一廊”绿色发展板块，扎实开展北沱河、奎濉河等重点河流水污染整治，大力防治畜禽养殖污染，划定畜禽养殖禁养区、限养区，全面推行市县乡三级“河长制”。启动采煤沉陷区生态修复治理，完成芦岭湖城市湿地规划编制。加强水源地保护，加大城乡黑臭水体整治。统筹抓好农业面源污染、土壤污染治理。落实市城镇绿化条例，启动林业增绿增效行动，造林16.8万亩。

统筹发展社会事业。推进主城区名校建分校，抓好义务教育均衡发展迎国检。继续实施学前教育行动计划，推进普通高中考试评价制度改革，加快现代职业教育体系建设，支持宿州学院等高等院校发展。完善公共文化设施网络，加强文化遗产保护与利用，广泛开展文化惠民活动，扶持戏曲、书画等文艺事业发展，提高文化供给能力。积极申办第十五届省运会。大力发展卫生计生事业，深入实施卫生人才队伍建设三大计划，加快建设标准化乡镇卫生院、标准化村卫生室，实施“四型医院”创建，开展“健康进万家”活动，积极推进“三保合一”，不断提升计划生育服务管理水平。全面落实各项就业创业政策，加强职业技能培训，强化困难人员就业援助。织密扎牢社会保障网，“五险”参保185万人次以上，城乡居民社会养老保险参保320万人以上。加快养老事业发展，完善保障老年人权益制度。积极打造孝文化名城、儿童幸福生活城、关爱弱势群体模范城。大力支持人民团体发挥桥梁纽带作用，认真做好民族宗教、外事侨务、防震减灾、残疾人、红十字、地方志、档案、对台、气象等工作。

积极创新社会治理。深化平安宿州建设，依法严厉打击各类违法犯罪行为。落实重大决策社会稳定风险评估、重大不稳定问题清单制度，集中化解信访积案。严格落实安全生产责任和管理制度，深入开展“铸安行动”。加强食品药品安全监管，深化食品药品安全示范城创建，加快建设市食品检验中心。全面完成城镇社区标准化建设，继续推进农村社区建设试点。优化应急联动和防灾减灾救灾机制。完善价格调控体系，保持市场平稳运行。扎实开展“七五”普法。加强国防动员、人民防空和国防后备力量建设，推进军民深度融合发展，常态化做好“双拥”工作。

扎实推进33项民生工程，继续办好10件惠民实事，以更大力度提供更多更好的公共服务。

1﹒主城区继续实施集中供热试点工程，5000户居民用上暖气。

2﹒主城区新增120辆新能源公交车、30个公交车充电桩，新建改建5个公交首末站，优化公交线网，建成公交智能调度和信息化管理系统。

3﹒对主城区12个黑臭水体进行综合治理。

4﹒主城区建成1500个免费Wi-Fi热点。

5﹒实施老城区道路、环城河、公园亮化工程。

6﹒完成主城区公厕改造提升。

7﹒实施农村饮水安全巩固提升工程，再解决90万人饮水安全问题。

8﹒智能化改造全市乡镇（街道）为民服务中心大厅，建设村（社区）政务服务全程代理工作站。

9﹒新建100个标准化村卫生室。

10﹒在农村偏远地区建设135个惠农金融服务室。

三、坚决打赢脱贫攻坚年度战役

认真贯彻精准扶贫、精准脱贫基本方略，聚焦脱贫攻坚十大工程，推进精准扶贫、监测评估、防范返贫、责任落实四项机制全覆盖，完成107个贫困村出列、12万人脱贫任务。

强化精准施策。继续实施贫困村整村推进工程、“一村一品”产业推进行动，引导贫困户参与新型农业经营主体生产经营活动，加强精准到户产业扶贫。推进就业脱贫工程，完成市级就业扶贫基地认定工作，实现贫困劳动者就业2000人。实施光伏脱贫工程，新建村级光伏电站70个、户用光伏电站1.1万户。发展电商扶贫，新建贫困村电商平台500个。发展乡村旅游扶贫，创建省级乡村旅游示范村、市级旅游扶贫示范点各5个。推进金融扶贫，加大扶贫小额信贷、贫困户子女助学贷款发放力度。完善各类教育资助方式，实现贫困学生资助全覆盖。落实健康脱贫政策，切实解决因病致贫、因病返贫问题。实施兜底脱贫工程，健全特困人员救助供养制度，实现低保线、扶贫线“两线合一”。实施易地扶贫搬迁2876人、危房改造12000户。加快补齐基础设施短板，续建新建农村道路畅通工程1500公里，逐步实现贫困村光纤宽带信息网络全覆盖。积极探索资产收益扶贫方式。

落实精准帮扶。进一步压实“单位包村、干部包户”、驻村扶贫工作队责任，加强基层组织和扶贫干部队伍建设，确保帮扶措施落实到位。实施“百企帮百村”精准扶贫行动，鼓励各类社会组织到村入户扶贫济困。深入开展扶贫日系列活动，促进社会资源向贫困人口聚集。坚持扶智强志，加强政策宣传和思想教育，不断激发脱贫内生动力，引导贫困户自力更生、自主脱贫。

完善体制机制。严把精准识别标准，强化动态管理，应退则退、应进则进，确保扶贫对象更加精准。落实防范返贫机制，对已脱贫人口、已出列贫困村，在一定时期内，保持原有扶贫政策不变、支持力度不减，确保稳定脱贫。加大资金投入，建立财政专项扶贫资金稳定增长机制，市、县两级财政均按照当年地方财政收入增量的20%以上，增列专项扶贫资金预算。市、县两级政府清理收回财政存量资金中可统筹使用部分，确保50%以上用于脱贫攻坚。进一步整合涉农项目资金，确保40%以上优先投向贫困地区和贫困人口。加大融资力度，充分利用农发行、国开行长期低息扶贫专项贷款。强化资金监管，建立完善财政扶贫资金绩效评价体系，开展事前、事中、事后全过程绩效监督。健全考核奖惩机制，坚持“月调度、月点评”，强化督查、审计、专项巡察。完善市县乡村四级脱贫攻坚责任制，严肃问责追责。

四、着力提升政府工作水平

落实五大发展行动计划，完成全年目标任务，对政府工作提出了新的更高要求。我们必须牢固树立“四个意识”特别是核心意识、看齐意识，切实加强自身建设，讲看齐、见行动，全面提升政府工作效能。

持续建设法治政府。坚持“法无授权不可为、法定职责必须为”，时刻做到心中有法、行必依法，全力推进法治政府建设。严格执行政府决策程序，不断提升依法科学民主决策水平。严格规范行政执法权，积极推进重大执法决定法制审核、执法全过程记录、执法文书规范化、执法公示制度。加强重点领域立法，全面实行规范性文件“三统一”。自觉接受人大法律监督、政协民主监督，切实办好人大代表议案和建议、政协建议案和委员提案，广泛听取人民团体意见，全面接受新闻媒体和人民群众监督。深入推进政务公开，确保权力在阳光下运行。

持续建设为民政府。坚持把群众获得感、社会满意度作为政府工作的晴雨表、风向标，全力以赴解民忧，全心全意惠民生。创新联系群众方法，完善市长热线、政风行风热线功能，多渠道了解社情民意、回应群众关切。加大社会事业领域政府购买服务力度，不断提高政府服务效率和质量。建设公共基础数据库和信息服务平台，推进政府部门信息共享。加强“互联网+政务服务”体系建设，优化政务服务流程和服务平台，促进实体政务大厅向网上办事大厅升级，最大程度方便群众和企业。

持续建设高效政府。切实强化担当意识，时刻把责任扛在肩上，恪尽职守，夙夜在公，坚决做到知责、尽责、负责。紧盯“动态指标进入全省前列”，深入推行“一线工作法”，严肃绩效管理和行政问责，建立健全正向激励和容错纠错机制，不断提高政府工作效率和质量。推进政府服务方式创新，勇于破除思维惯性和路径依赖，善于运用市场化、社会化方式，创造性地开展各项工作。推动效能建设常态化、长效化，重拳整治“不作为、乱作为、慢作为”、“中梗阻、基层板结”，切实打通“最先最后一公里”，确保政府工作掷地有声、落实见效。

持续建设廉洁政府。严格落实全面从严治党主体责任，全面加强和规范党内政治生活，自觉加强和接受党内监督，提升政府系统从严管党治党水平。全面落实党风廉政建设责任制和“一岗双责”，深入推进惩治和预防腐败体系建设。自觉践行“三严三实”，深入开展“讲看齐、见行动”学习讨论，巩固“两学一做”学习教育成果，严格执行中央八项规定精神和省市“30条”，继续抓好“四风”新问题治理。进一步规范政府采购、招标行为，加强“三重一大”事项管理。推动审计监督全覆盖，对重大项目、重点工程实行全过程监督。强化行政监察，加大违纪违法案件查处力度，严肃责任追究，以廉政建设和反腐败斗争的新成效，保障发展、取信于民。

各位代表！实干成就梦想，拼搏创造未来。让我们紧密团结在以习近平同志为核心的党中央周围，在市委的坚强领导下，凝心聚力、锐意进取，不忘初心、继续前进，加快建设五大发展的美好宿州，以优异成绩迎接党的十九大胜利召开。
